# Supplementary material for: Ten-valley excitonic complexes in charge-tunable monolayer WSe2
Source: Nat Commun. 2025 Nov 5;16:9743. doi: 10.1038/s41467-025-65731-x (PMC12589553; doi:10.1038/s41467-025-65731-x)
Supplement: Supplementary file 1 — Supplementary Information [file 41467_2025_65731_MOESM1_ESM.pdf]

Supplementary Information for: Ten-valley excitonic complexes in charge-tunable monolayer WSe_2

Alain Dijkstra,[1,2,3,*] Amine Ben Mhenni,[1,2,3,*] Dinh Van Tuan,[4] Elif Çetiner,[1,2,3] Muriel Schur-Wilkens,[1,2,3] Junghwan Kim,[4] Laurin Steiner,[1,2,3] Kenji Watanabe,[5] Takashi Taniguchi,[6] Matteo Barbone,[1,2,3] Nathan P. Wilson,[1,2,3] Hanan Dery,[4,7] and Jonathan J. Finley[1,2,3]

[1] *Walter Schottky Institute, Technical University of Munich, 85748 Garching, Germany*
[2] *Technical University of Munich, TUM School of Natural Sciences, Physics Department, 85748 Garching, Germany*
[3] *Munich Center for Quantum Science and Technology (MCQST), Schellingstr. 4, 80799 München, Germany*
[4] *Department of Electrical and Computer Engineering, University of Rochester, Rochester, NY, United States.*
[5] *Research Center for Electronic and Optical Materials, National Institute for Materials Science, 1-1 Namiki, Tsukuba 305-0044, Japan*
[6] *Research Center for Materials Nanoarchitectonics, National Institute for Materials Science, 1-1 Namiki, Tsukuba 305-0044, Japan*
[7] *Department of Physics and Astronomy, University of Rochester, Rochester, NY, United States.*

LIST OF FIGURES

1 **Gate-dependent optical response of WSe_2 control sample.** 2
2 **Fitting of the gate-dependent reflection contrast of the main WSe_2 device.** 3
3 **Dispersion of Landau levels in WSe_2.** 4
4 **Valley population calculations versus band energies in WSe_2.** 5
5 **Magneto-Photoluminescence of WSe_2.** 6

* These authors contributed equally to this work.

✉ email: Alain.Dijkstra@tum.de; Amine.Ben-Mhenni@tum.de; J.J.Finley@tum.de

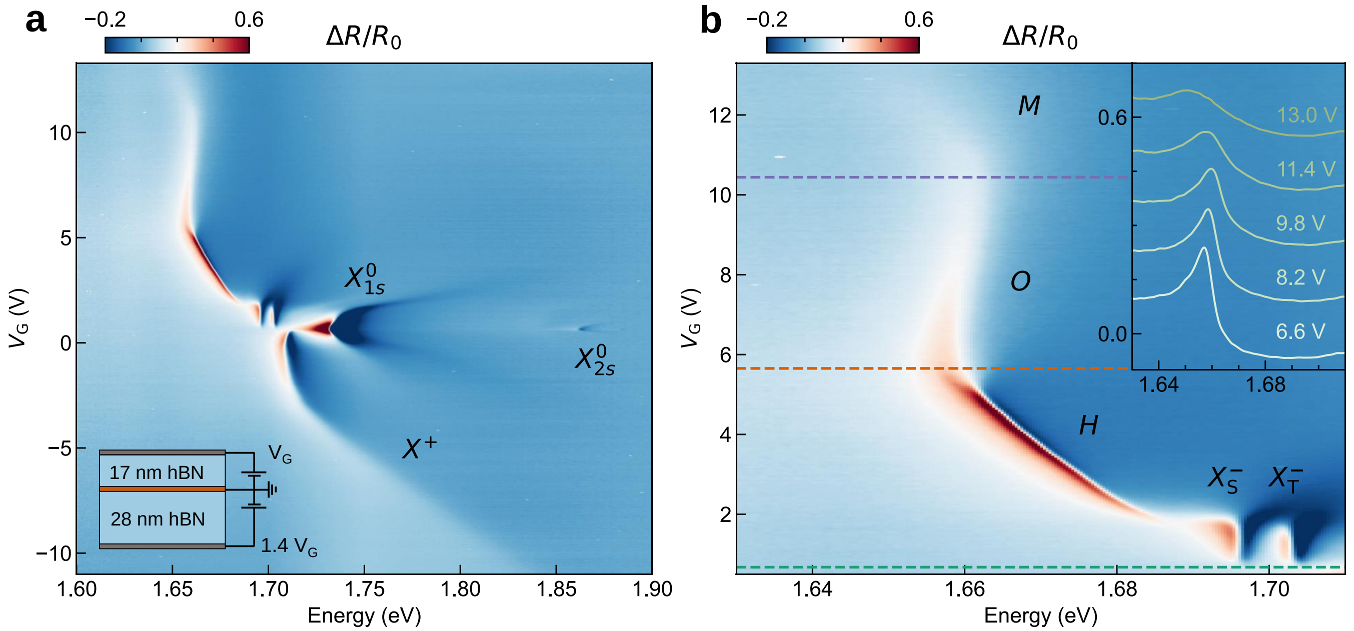

Supplementary Figure 1. **Gate-dependent optical response of WSe₂ control sample.**
a, Gate-dependent reflection contrast spectra of our control WSe_2 device recorded at 4 K, the inset shows a schematic of the dual-gated WSe_2 device with labelled hBN thicknesses. Due to the asymmetry in the hBN spacer thicknesses the bottom gate can be biased more than the top gate. Therefore the voltage on the y-axis is the voltage applied to the top gate, where the voltage at the bottom gate is $V_b = 1.4 \cdot V_t$. **b,** Close-up view of the negatively charged regime from (a) revealing the same excitonic complexes as observed in Fig. 1 of the Main Text: the exchange-split trions ($X^-_{S,T}$), the hexciton (H), the oxciton (O), and the many-body complex (M). The start of the filling of the lower K/K' valleys, the upper K/K' valleys, and the Q/Q' valleys are marked with a green, orange, and purple dashed line, respectively. Notably, the energy at which the Q/Q' valleys reside (Δ_{KQ}) in this control sample, is different from the value of the main sample shown in Fig. 2a of the Main Text. This is evidenced by the different ratios of the electron densities at which O appears (filling of the upper K/K' valleys) and the density at which M appears (filling of the Q/Q' valleys). This ratio is given by $(V_Q - V_0)/(V_{uK} - V_0)$, in which V_0, V_{uK} and V_Q, are the voltages at which the filling starts of the lower valleys at K/K', the upper valleys at K/K' and the valleys at Q/Q' respectively, which yields 1.81 and 1.96 for the main sample shown in Fig. 2a of the Main Text and the control sample shown here respectively. Filling in these ratios in Supplementary Data Fig. 4c an ∼8 % increase in Δ_{KQ} is found for the control sample with respect to the main sample. We attribute this difference to the different dielectric environments due to different hBN thicknesses, which influences the Q/Q' valleys stronger than the K/K' valleys.

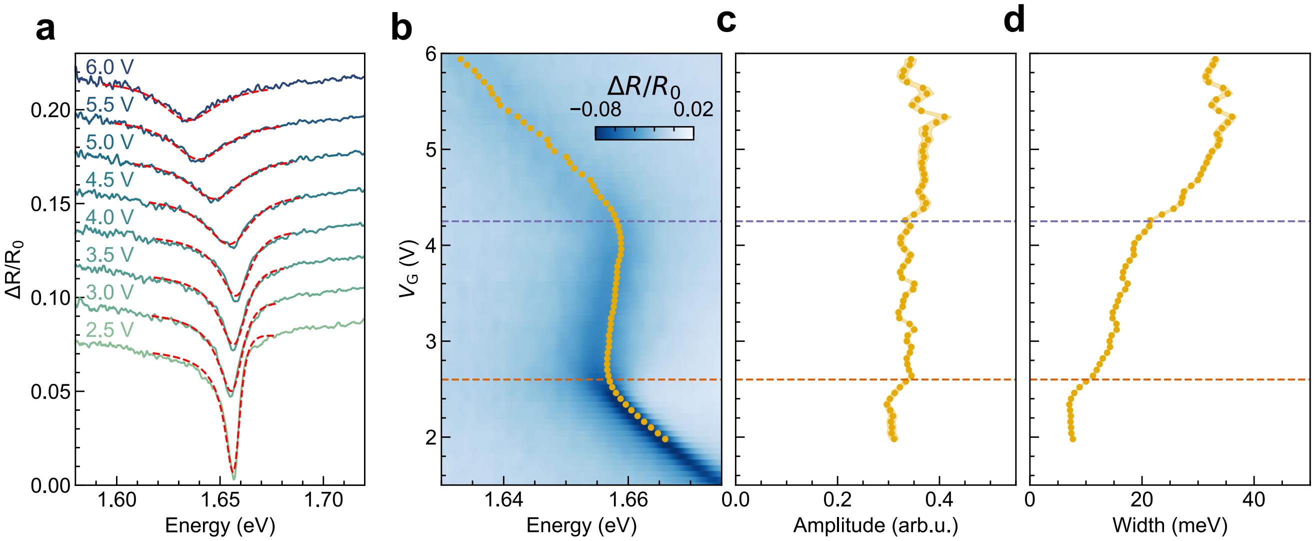

Supplementary Figure 2. **Fitting of the gate-dependent reflection contrast of the main WSe_2 device.** Low-temperature reflection contrast data, presented in Fig. 1 of the Main Text, are fitted using a dispersive Lorentzian function (see Methods) for the voltage range of 2 V to 6 V making fitting with a single dispersive Lorentzian feasible. In this range, the hexciton (H), the oxciton (O), and the many-body complex (M) are observed without spectral overlap of other resonances. **a,** Example reflection contrast spectra with the corresponding fits overlayed as dashed red lines. **b,** A heatmap of the recorded reflection contrast spectra with the center energies of the dispersive Lorentzian fits plotted in dark yellow on top. The fits accurately follow the recorded resonances and confirm the red-shift, blue-shift, red-shift character of the H, O, and M excitons, respectively. The orange (purple) horizontal dashed line marks the transition from H to O (O to M) exciton. **c,** The amplitude of the resonance resulting from the fits. The shaded region represents the fitting error as given by the least-squares method. Importantly, we observe no significant change in the amplitude for any of the three resonances. **d,** The full width at half maximum of the resonances resulting from the fits. We observe an almost constant width for the H exciton, which agrees with the composite excitonic states model, because it is an optimal complex with a distinct photoexcited electron-hole pair. Starting from the transition from H to O, we observe a continuous broadening that also extends to the M exciton. In addition there are steps in the width of the resonance when transitioning from H to O and from O to M. These observations are expected for O and M, as they are complexes with an indistinct photoexcited electron-hole pair.

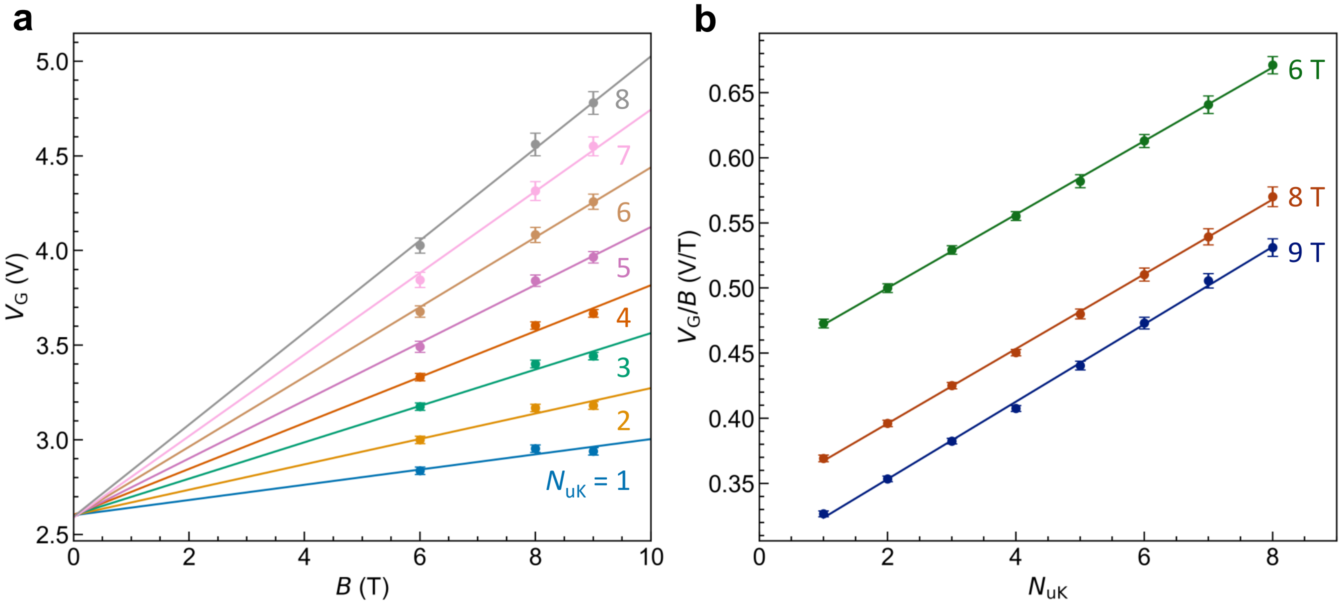

Supplementary Figure 3. **Dispersion of Landau levels in WSe_2.**
a, Dispersion of the first 8 Landau levels determined from σ^+ polarized data (photoexcited electron-hole pair associated to the K valley) for 6 T, 8 T and 9 T magnetic field. Each data point marks the disappearance of a resonance due to Pauli blocking in regime III as plotted in Fig. 3a of the Main Text. The error bars represent either the resolution of the measurement or the accuracy with which the levels could be distinguished, whichever value is greater. **b,** Contains the same data as (a) but now represented as V_G/B versus the index of each Landau level in the upper K/K' CB (N_{uK}) for different B-fields. In this plot, the slope directly gives a value for $\Delta V/B$ (ΔV being the voltage interval between two disappearing Landau levels), which is the main parameter to calibrate the charge density (see Methods). Averaging the slopes determined from the curves for the three magnetic fields gives $(28.7 \pm 0.2)\,\mathrm{mV\,T^{-1}}$.

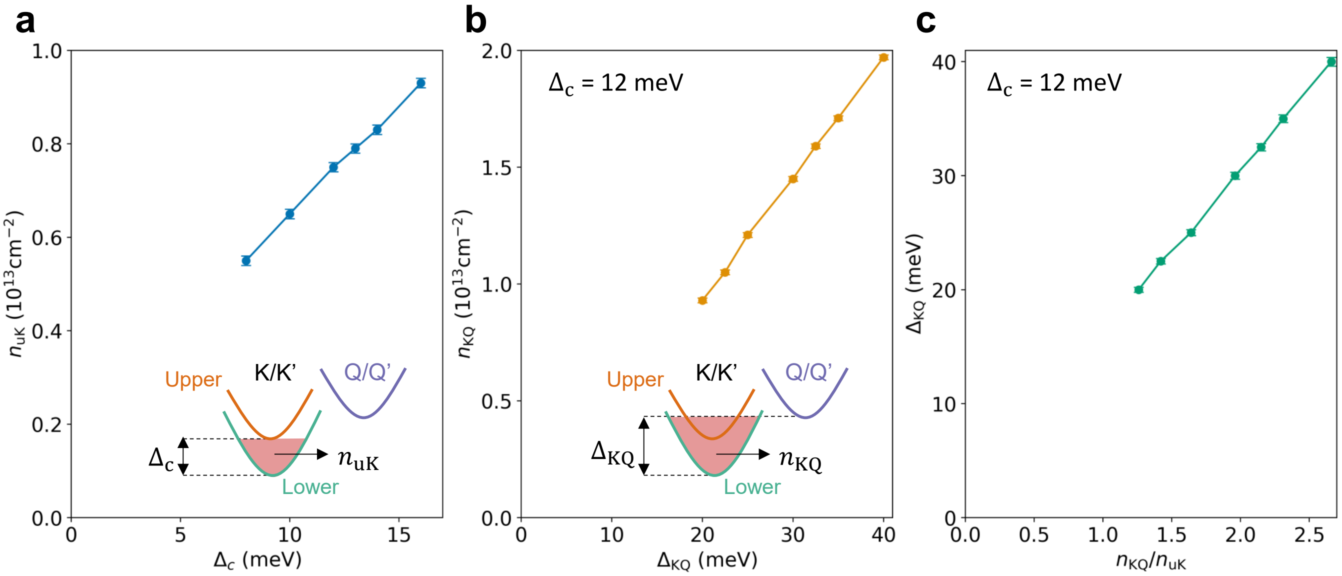

Supplementary Figure 4. **Valley population calculations versus band energies in WSe_2.** Using the valley population model as described in the Methods we have performed calculations in which the carrier density of each valley is determined as a function of the overall electron density (n_T) and therefore predicts at which n_T, the different valleys start filling. This model takes effective masses, interaction parameters, and the energy intervals between the band-minima (Δ_x) as input parameters. For all calculations we have used $m_l = 0.4m_0$, $m_u = 0.29m_0$ and $m_Q = \sqrt{0.45 \cdot 0.75}m_0$ for the effective mass of the lower CB at K/K', the upper CB at K/K' and the Q/Q' valleys respectively based on ref.[1]. **a,** We run our model iteratively while varying the energetic spacing between the lower and upper CB K/K' valleys (Δ_c) and record the density at which the upper K/K' valleys start filling (n_{uK}). We find an approximately proportional relationship between the two parameters. For a value of $n_{uK} = 0.8 \times 10^{13}\,\mathrm{cm}^{-2}$, determined from the calibration shown in Fig. 2a of the Main Text, a value of $\Delta_c = 13\,\mathrm{meV}$ is found. This value not only closely agrees with the literature values [2, 3] of 12 - 14 meV, but also shows the validity of both our charge density calibration strategy as well as the valley population model. The inset shows a cartoon of the band structure representing the point at which the upper K/K' valley starts to fill. **b,** To estimate the energy interval between the lower K/K' valleys and the Q/Q' valleys (Δ_{KQ}) we run the model iteratively, varying Δ_{KQ} while calculating the density at which the Q/Q' valleys start filling (n_{KQ}) and keeping Δ_c at a constant value of 12 meV. Our calibration shown in Fig. 2a of the Main Text yields a value of $n_{KQ} = 1.5 \times 10^{13}\,\mathrm{cm}^{-2}$, which allows us to determine $\Delta_{KQ} = 30\,\mathrm{meV}$. The inset shows a cartoon of the band structure representing the point at which the Q/Q' valleys start to fill. **c,** A plot of Δ_{KQ} as function of the ratio of the threshold charge densities n_{KQ}/n_{uK}, while keeping Δ_c at $\sim$12 meV, based on the same calculated data shown in (b). This representation is useful because it allows one to determine Δ_{KQ} without an absolute calibration of n_T. The ratio n_{KQ}/n_{uK} relates to experimental parameters directly as $n_{KQ}/n_{uK} = (V_Q - V_0)/(V_{uK} - V_0)$, in which V_0, V_{uK} and V_Q, are the voltages at which the filling starts of the lower K/K' valleys, the upper K/K' valleys and the Q/Q' valleys respectively. All error bars represent the resolution with which the calculation was performed, marking a minimum value for the error.

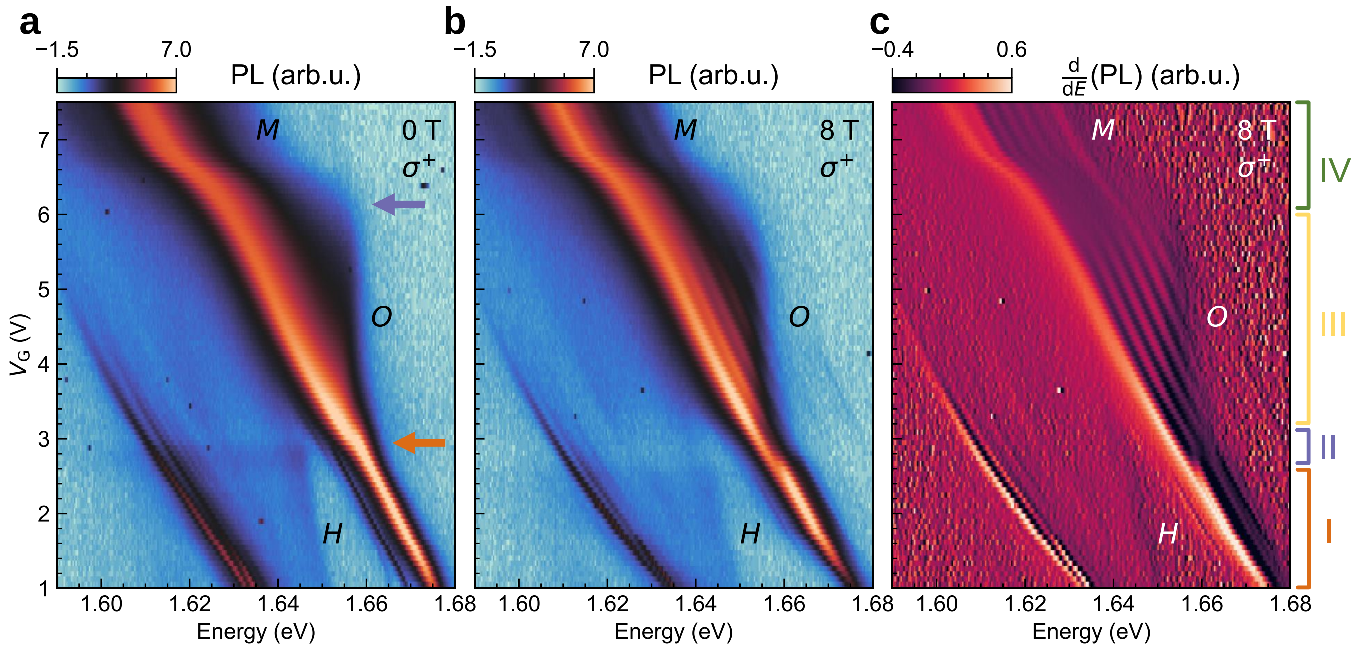

Supplementary Figure 5. **Magneto-Photoluminescence of WSe_2.**
a, Gate-dependent photoluminescence (PL) measurement of the main device measured at a mixing chamber temperature of ∼20 mK, presented on a logarithmic scale. The gating range is chosen to highlight the H, O, and M resonances. The back gate of the double-gated sample is kept at 2 V while the voltage of the top gate V_G is swept from 2 V to 7.5 V. At ∼2.9 V the filling of the upper K/K' valleys starts with the change of H to O, marked by the onset of a gradual asymmetric broadening of the PL signal for increasing voltage (orange arrow). At ∼6.4 V (purple arrow), the filling of the Q/Q' valleys is signalled with the onset of a sudden increased rate of redshift of the maximum of the PL signal, together with a change in broadening. **b,** The same experiment as in (a), but in an 8 T magnetic field with co-polarized, σ^+ excitation and detection. **c,** The derivative with respect to energy of the data shown in (b). This representation highlights the oscillations in the PL signal and shows the Landau level resonances. On the far right, colored brackets indicate different doping regimes for panels (b) and (c), corresponding to the bandstructure drawings in Fig. 3d of the Main Text. The H exciton shows almost no additional resonances that resemble Landau levels. We understand this because for a distinguishable photoexcited electron-hole pair, all photoexcited electrons can relax to the lowest Landau level in the upper K/K' valley before recombination. Then, for O progressively more resonances appear with increasing charge density, building up a fan of Landau levels. This behavior is opposite to the O observed in reflection contrast shown in Fig. 3 of the Main Text, where resonances are progressively disappearing. Interpreting these observations, we phenomenologically conclude that reflection contrast measurements are sensitive to Landau levels in the upper K/K' valleys above the Fermi level, and PL is sensitive to Landau levels in the upper K/K' valleys below the Fermi level. We notice that the M exciton, contrasting to reflection contrast, also shows Landau level resonances without additional resonances appearing for increasing charge density. For an emission process, the photoexcited electron can reside in a Landau level in the upper K valley, but below the onset of the Q/Q' valley. Such an electron does not suffer from intervalley scattering. We argue that any photoexcited electron residing in a Landau level above the onset of the Q valley is subject to intervalley scattering and will show dephasing and therefore broadening, such that the individual Landau levels are not resolvable.

SUPPLEMENTARY REFERENCES

[1] A. Kormányos, G. Burkard, M. Gmitra, J. Fabian, V. Zólyomi, N. D. Drummond, and V. Fal'ko, $\mathbf{k} \cdot \mathbf{p}$ theory for two-dimensional transition metal dichalcogenide semiconductors, 2D Materials **2**, 022001 (2015).

[2] L. Ren, C. Robert, H. Dery, M. He, P. Li, D. Van Tuan, P. Renucci, D. Lagarde, T. Taniguchi, K. Watanabe, X. Xu, and X. Marie, Measurement of the conduction band spin-orbit splitting in WSe_2 and WS_2 monolayers, Physical Review B **107**, 245407 (2023).

[3] P. Kapuściński, A. Delhomme, D. Vaclavkova, A. O. Slobodeniuk, M. Grzeszczyk, M. Bartos, K. Watanabe, T. Taniguchi, C. Faugeras, and M. Potemski, Rydberg series of dark excitons and the conduction band spin-orbit splitting in monolayer WSe_2, Communications Physics **4**, 186 (2021).
